# RNA Editing and Modifications in Mood Disorders

**DOI:** 10.3390/genes11080872

**Published:** 2020-07-31

**Authors:** Alessandro Barbon, Chiara Magri

**Affiliations:** Section of Biology and Genetics, Department of Molecular and Translational Medicine, University of Brescia; Viale Europa 11, 25123 Brescia, Italy; chiara.magri@unibs.it

**Keywords:** RNA editing, ADAR1, ADAR2, major depressive disorders, psychiatric disorder, depression, stress, antidepressant, m6a methylation

## Abstract

Major depressive disorder (MDD) is a major health problem with significant limitations in functioning and well-being. The World Health Organization (WHO) evaluates MDD as one of the most disabling disorders in the world and with very high social cost. Great attention has been given to the study of the molecular mechanism underpinning MDD at the genetic, epigenetic and proteomic level. However, the importance of RNA modifications has attracted little attention until now in this field. RNA molecules are extensively and dynamically altered by a variety of mechanisms. Similar to “epigenomic” changes, which modify DNA structure or histones, RNA alterations are now termed “epitranscriptomic” changes and have been predicted to have profound consequences for gene expression and cellular functionality. Two of these modifications, adenosine to inosine (A-to-I) RNA editing and m6A methylations, have fascinated researchers over the last years, showing a new level of complexity in gene expression. In this review, we will summary the studies that focus on the role of RNA editing and m6A methylation in MDD, trying to underline their potential breakthroughs and pitfalls.

## 1. Introduction

Major depressive disorder (MDD) is characterized by symptoms such as profound sadness, a decrease in vitality, loss of interest in normal activities and negative and pessimistic thoughts. It is a very frequent and widespread disorder. The World Health Organization (WHO) evaluates MDD as one of the most disabling disorders in the world with a lifetime prevalence ranging from 12% to more than 20% and with a very high social cost [1]. MDD is a multifactorial disorder due to the additive effects of hundreds of susceptibility genes and environment factors, interacting with each other in predisposed individuals causing the development of the illness. A recent genome-wide association (GWA) meta-analysis of MDD has identified 102 single nucleotide polymorphisms (SNPs) mapping in 269 genes associated with depression [2,3].

Beside genetic variations, other alterations that might underlie the pathological behaviors observed in depressed individuals are stress-induced epigenetic modifications [4]. Indeed, alterations of DNA methylation, histone organization and noncoding RNAs have been found to be associated with both depression and depression-like behaviors induced by stress in animal models [5]. Until now, however, little attention has been given, in the context of MDD, to understand the role of RNA modifications in modulating gene expression.

Recently, “omics” approaches have revealed that RNA molecules are broadly and dynamically altered by many different mechanisms. More than 100 RNA modifications have been identified [6]. Similar to “epigenomic” changes, which alter DNA structure or modify histones, these RNA alterations have been termed “epitranscriptomic” changes [7,8] and are predicted to impact both gene expression and cellular functionality [9].

One of the most studied processes occurring at the RNA level is the co-transcriptional event of “RNA editing” [10]. The first example of “RNA editing” was described in the mitochondrial RNA of trypanosomes and consisted in the insertion of uridines [11]; now, the term “RNA editing” refers to any RNA site-specific substitution. RNA editing can modify the primary structure of the proteins by introducing single amino acid substitutions, new start and stop codons or by modifying splicing sites. Furthermore, it can also affect RNA stability by modifying the Untranslated Regions (UTRs) [12,13].

In addition to RNA editing, RNA molecules are altered by a variety of reversible chemical modifications [6]. Among these, adenosine methylation at position N6 (namely N6-Methyladenosine -m6A) is one the most profuse in eukaryotic mRNAs. Recent studies revealed that the adenosine methylation at position N6 is dynamically regulated by three families of enzymes namely writers, erasers and readers [8,14]. The level of methylation seems to affect the processing of mRNAs, their translation and decay, and to play an important role in particular during embryonic development and stress response. Despite the biological role of m6A, its impact is still largely unexplored in psychiatric conditions.

In this revision, we will try to summarize the state of art of the studies that focus on the role of RNA modifications in MDD trying to underline their potential breakthroughs and pitfalls.

## 2. Adenosine to Inosine (A-to-I): The Most Common Form of RNA Editing

Adenosine to inosine (A-to-I) editing is the most frequent form of RNA editing, widespread in the majority of human genes. The conversion of adenosine to inosine is mediated by enzymes named Adenosine Deaminases Acting on RNA (ADAR); ADAR enzymes are able to bind to double stranded RNA (dsRNA) and modify A nucleotide into I by deamination. The mammalian genome encodes for three members of the ADAR family: ADAR1 (encoded by the gene ADAR), ADAR2 (encoded by ADARB1) and ADAR3 (encoded by ADARB2). ADAR1 and -2 are active enzymes, whereas ADAR3 lacks the enzymatic activity and is mainly expressed at low-level in brain regions [15,16]. ADAR1 has two splicing isoforms: the constitutive 110-kDa (p110) and the interferon-inducible (p150) isoforms [17]. ADAR2 have several splicing isoforms, among which the two more abundant are: the short ADAR2S (ADAR2a) and the long ADAR2L (ADAR2b), which differ due to alternatively splicing on the deaminase domain, leading to distinct functional activity [18,19].

ADAR enzymes edit millions of sites in the mammalian transcriptome. The majority of these sites is located in non-coding sequences: 5′UTRs, 3′UTRs and in intronic retrotransposons, such as ALU-inverted repeats [20]. In principle, RNA editing in repetitive elements, such as ALU sequences, is primarily mediated by ADAR1, whereas RNA editing at the coding sites (namely re-coding sites) is preferentially performed by ADAR2; however, a certain degree of overlap exists between target sites of the two enzymes [21]. About 2.5 million editing sites have been identified up to now and are deposited in public databases [22,23].

Many re-coding sites are phylogenetically conserved and affect transcripts important for neuronal functions [24]. For example, permeability of ion channels and responses to excitatory neurotransmitters have been found to be altered by RNA editing of re-coding sites [25]. In addition, in vivo and in vitro studies have shown that these sites are strongly regulated throughout development [26,27]. Due to the involvement of RNA editing in the correct cellular functions of the brain and in neurodevelopment, the role of RNA editing in psychiatric disorders has gained an increasing interest. At present, RNA editing had been reported in several neurological and psychiatric disorders, including major depression (see the following paragraphs), Alzheimer’s disease [28,29], amyotrophic lateral sclerosis [30], schizophrenia [31] and autism [32,33]. However, due to the lack of transcriptome-wide analyses of RNA-editing sites for the majority of psychiatric disorders, how pervasive altered RNA editing is in the brain of psychiatric patients is still not clear.

### 2.1. RNA Editing in MDD

Over the decades, RNA editing studies in MDD have focused mainly on the analysis of the serotonin 2C receptor (5-HT2c-R) and glutamate receptor transcripts, in line with the serotonergic and glutamatergic hypothesis of mood disorders [34,35].

#### 2.1.1. RNA Editing in the Serotonin Receptor 2C

**Figure 1:**
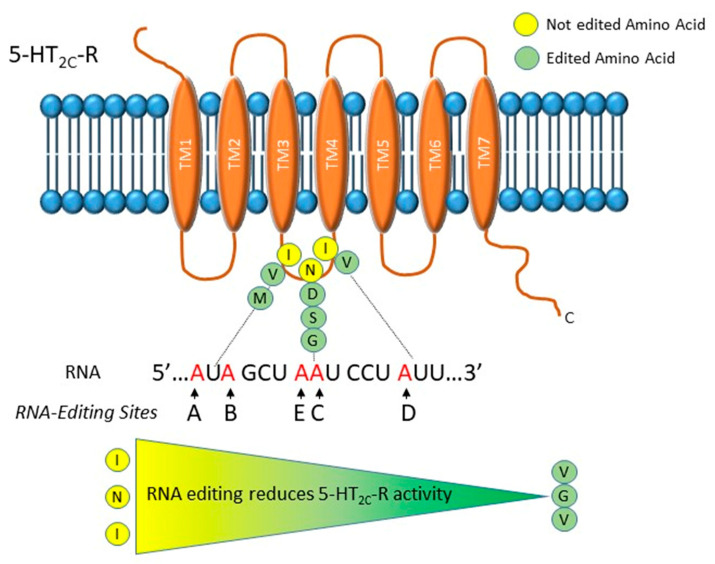
Graphical representation of the serotonin 2C receptor (5-HT2c) receptor and its editing sites. The five editing sites, named A, B, C, D and E, are mapped in the RNA sequence that encodes for the second intracellular loop of the receptor. At the protein level, they induce one to three amino acids changes that can result in twenty-four different protein isoforms with different G-protein-coupling activity. In particular, it has been reported that the edited isoforms have a decreased G-protein-coupling activity and in turn a lower serotonin affinity. Thanks to its ability of modulating serotonin signaling, dysregulation in 5-HT2c receptor RNA editing might be involved in the etiology of major depressive disorder (MDD).

The 5-HT2c-R is a G-protein coupled receptor inducing the activation of phospholipase C and the generation of inositol phosphates and diacylglycerol [36,37]. This receptor is modified by RNA editing that generates protein forms with different functionality [38]. In particular, in the sequence that codes for the second intracellular loop of the receptor, five adenosines, termed A, B, C, D and E, undergo A-to-I RNA editing (Figure 1). These modifications induce conformational changes that decrease 5-HT2c-R’s G-protein-coupling activity, agonist affinity and in turn serotonin signaling [38]. The A, B and C sites are edited by ADAR1, while editing at the D site is done by ADAR2 [39]. Both ADAR1 and ADAR2 might edit the E site (former C′). Combinations of RNA editing events of the five sites cause one to three amino acids changes that can result in 24 different protein isoforms, going from the unedited (INI) to the completely edited (VGV) form. Edited receptors show reduced G protein coupling and reduced affinity for serotonin (Figure 1) [38].

The main studies tackling the association between MDDs and RNA editing in the serotonin receptor 2C are summarized in Table 1. One of the first analysis of the 5-HT2c-R RNA editing in the prefrontal cortex (PFC) of subjects with MDD, revealed no significant variations in RNA editing compared to control subjects and to schizophrenia patients. However, suicide victims (regardless of diagnosis) showed an increased editing level at the A site [40]. The increased level of editing at the A site in suicide victims was also confirmed by Iwamoto et al. [41]. One work reported that MDD patients that committed suicide showed increased editing at the E site and reduced editing at the D site, while the C site showed a trend of increase [42]. Interestingly, mice treated with the antidepressant drug fluoxetine, a Selective Serotonin Reuptake Inhibitor (SSRI), showed modifications in the editing level of the E, C and D sites that were opposite to those observed in suicide victims [42]. However, another study showed no variation in editing level of D site in suicide victims who suffered of MDD, but a trends of increased RNA editing in MDD subjects [41]. In recent years, several studies analyzed 5-HT2c-R-edited isoforms through next-generation sequencing approaches. Zhu et al. [43] showed no significant modifications of RNA editing of 5-HT2c-R in the brain of MDD patients but a trend of reduced editing at the C, D and E sites in non-suicidal depression. Other studies reported of higher editing levels in PFC of MDD patients who committed suicide compared to those who died of different causes [44,45]. Recently, modifications of editing levels of 5-HT2c-R mRNA were observed not only in PFC (Brodmann Area 9) but also in anterior cingulate cortex (Brodmann Area 24) of depressed suicide victims. Specifically, a robust increase in editing on 5-HT2c-R was seen in the anterior cingulate cortex of suicide victims, linking the editing reaction in this cortical area to suicide risk [46]. Intriguing, phosphodiesterases (PDE), a key modulator of signal transduction downstream 5-HT2c-R, involved in inflammatory cell activation, memory and cognition, was differently edited between depressed suicide decedents and controls [47].

As already noted [48], Table 1 clearly indicates that literature data is not always consistent, which could be due to different factors such as the number of samples analyzed, inconsistency in the precise brain regions analyzed, the techniques used to measure editing levels, technical artefact associated to post-mortem brain etc. Despite these discrepancies, the most recurrent result is a trend of increased editing in depressive patients that committed suicide. This might indicate that suicide is associated with higher level of 5-HT2c-R editing, and thus lower receptor activity, mainly in the PFC [45].

Finally, the link between serotonin transmission, 5-HT2c-R editing and the risk of suicide is further strengthen by the observation that polymorphisms located in the ADARB1 (coding for ADAR2) and HTR2C genes where associated with suicidal attempts [49] and ADARB1 and Tryptophan hydroxylase 2 (TPH2) polymorphisms with suicide attempts after general childhood traumas [50].

#### 2.1.2. RNA Editing in Glutamate Receptor Transcripts

**Figure 2:**
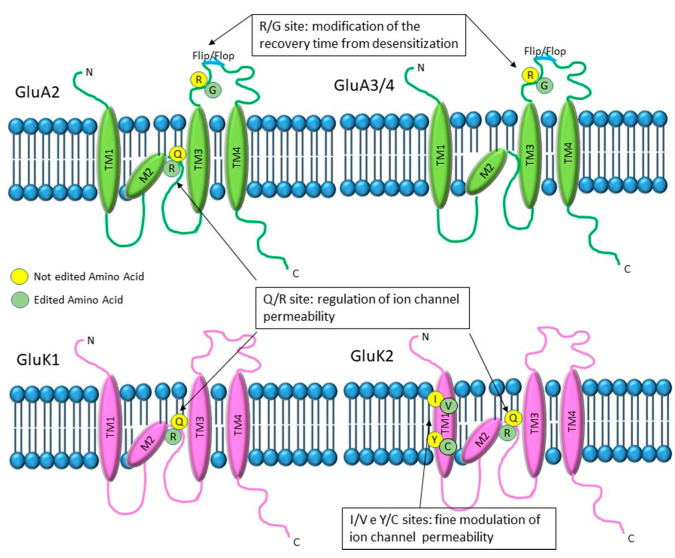
Graphical representation of the alfa-Amino-(3-Idrossi-5-Metil-4-isoxazole) propionate (AMPA) and Kainate glutamate receptors and their editing sites. GluA2/3/4 AMPA subunits have an edited site that converts arginine to glycine (R/G) located in the extracellular loop adjacent to the neurotransmitter binding site. Editing at this site enhances the recovery time from desensitization. In addition, GluA2 subunit and kainate GluK1 and GluK2 have a Q/R editing site, located in the second membrane domain, inside the channel pore. The Q/R site is involved in the regulation of ion channel permeability and is always fully edited in GluA2, whereas it shows variable levels of editing for the kainate receptor subunits. Finally, the GluK2 subunit presents two additional editing sites (I/V and Y/C) localized in the first transmembrane domain and involved in the fine tuning of ion channel permeability.

RNA editing modifies three alfa-Amino-(3-Idrossi-5-Metil-4-isoxazole) propionate (AMPA) (GluA2, GluA3, GluA4) and two kainate (GluK1 and GluK2) glutamate receptor subunits in different positions (Figure 2). In particular, all three AMPA subunits have an edited site that converts arginine to glycine (R/G) localized in the extracellular loop adjacent to the neurotransmitter binding site, before the flip or flop box sequence. In addition, the GluA2 subunit also has a Q/R site, converting a codon coding for glutamine to one coding for arginine, located in the second membrane domain located inside the channel pore. The kainate subunit GluK1, instead, has only the Q/R site inside the channel pore, whereas GluK2 presents two additional sites: at the I/V site, isoleucine is changed into valine, and at the Y/C site, a tyrosine is changed into a cysteine. Both sites are localized in the first transmembrane domain [51,52].

Research focusing on glutamate receptor RNA editing dysregulation in human post-mortem brains of MDD patients is lacking, or positive results are lacking. At present, only one study reported a modest decreased of R/G editing levels of AMPA receptors in patients with mood disorders, correlated with the downregulation of ADAR2 expression [53].

### 2.2. Stress Paradigm Affect RNA Editing Activity

Physical and psychological distress, if repeated, strongly affects genetically predisposed subjects and might be a main risk factors for neuropsychiatric disorders such as MDD [54]. Progresses in understanding the molecular mechanisms underpinning the role of stress on neuropsychiatric pathology come from the study of animal models subjected to different type of stress paradigms (i.e., learned helplessness, social defeat, chronic mild stress, foot shock stress paradigms). The same models are also useful tools for investigating the effect of behavioral stress on RNA editing.

As for human studies, the first reports correlating modifications in the editing reaction with stress events focused on the analysis of specific and well-known re-coding sites, such as those modulating serotoninergic [55] and glutamatergic neurotransmissions [52].

Many studies reported that common stress paradigms [56,57,58,59] alter RNA editing status of 5-HT2c-R. Nevertheless, the modifications observed were not consistent among studies. The use of mice genetically altered to express either the INI (unedited) or the VGV (completely-edited) 5-HT2c-R isoform, that differ in their sensibilization time and receptor signaling, allowed investigating the significance of 5-HT2c-R editing in the field of stress-related disorders [60]. Intriguing, both INI and VGV mice exhibit high anxiety-like behaviors in the elevated-plus maze paradigm [61]. INI isoform was associated with increased time being immobile in the forced-swim test (FST) and tail suspension test (TST). On the contrary, VGV isoform was associated with a decreased time being immobile, showing antidepressant-like activity in FST and TST [61]; it is of note that different results are reported for mice with different genetic backgrounds [61]. The VGV form also shows anxiogenic and aggressive behaviors, with altered 5-HT2c-R signaling within specific brain areas [62]. Furthermore, knock-in of the Htr2c gene generating ‘INI’ isoform show reduced depressive-like and fear-associated behaviors without altering anxiety behavior [63]. It has been recently shown that VGV mice present augmentation of both innate and conditioned fear and may be studied as a model for posttraumatic stress disorder (PTSD) predisposition [64]. These reports, with some discrepancy, clearly point to a role of 5-HT2c-R editing in mood and anxiety disorders and suggest that increased editing might cause stress-related disorders.

The first report linking glutamate receptor editing modifications and stress took advantage of fear conditioning, a behavioral test in which animals are able to foresee aversive stimuli. This stress paradigm induced a decrease in the RNA editing level of kainate GluK1 Q/R site in the amygdala [65]. Furthermore, the authors reported experience-dependent changes in mRNA expression of both ADAR2 and ADAR1 in the amygdala and hippocampus (HI), concluding that modifications in editing levels might play a role in experience-dependent synaptic modifications. Interestingly, it has been reported that ADAR2 +/- knockout mice showed increased activity in the open-field test together with resistance to immobility in the FS test and enhanced hyperactivity due to amphetamine intake: this behavioral phenotype was related to a decreased editing AMPA receptor R/G sites [53]. The authors suggest that in the onset of stress-related mental disorders, altered AMPA receptor RNA editing efficiency due to ADAR2 down-regulation might have a role. However, acute foot shock stress or chronic mild stress paradigms does not alter glutamate RNA editing for Q/R and R/G editing sites of GluA2, GluA3 and GluA4 AMPA subunits in the PFC [66] or HI of treated animals [67]. Further studies are needed to clarify the role, if any, of glutamate receptor RNA editing in stress paradigms.

As previously mentioned, for a long time the analysis of changes in the RNA editome remained restricted to re-coding sites. Recently, thanks to Next Generation Sequence (NGS) technology, the analysis of RNA editing of a large number of sites located throughout the transcriptome has become feasible and allowed having a more detailed picture of the involvement of RNA editing in stress and stress-related disorders. A recent work analyzing chronic social defeat stress (CSDS) quantified RNA editing at hundreds of sites, using a microfluidics-based multiplex polymerase chain reaction and a deep sequencing (mmPCR-seq) analysis revealed that CSDS induced moderate changes in RNA editing in a set of mRNAs within the Basal lateral amygdala (BLA) and medial PFC, including modest alterations of the 5-HT2c-R transcript [68]. The authors hypothesized that such modest modifications of RNA editing might be likely explained by cellular heterogeneity of the brain sample tissues used. This hypothesis has been confirmed by recent advances in transcriptomic analyses of single cells, demonstrating that RNA editing is likely cell-specific and changes might be observed between different type of cells within the brain [69,70].

Finally, it has been reported that if adolescent female rats are exposed to chronic unpredictable stress ahead of reproduction (also called pre-reproductive stress: PRS), RNA editing is affected in the PFC and amygdala of the offspring for two generations. The editing level of the five 5-HT2c-R sites resulted to be affected by stress and led to the expression of different 5-HT2c-R isoforms in stress-exposed offspring versus naïve females [71]. In particular, the stress paradigm induced increased editing levels at the A site in PFC of adolescent female, whereas editing of A and B sites decreased in the first filial generation but increased in the second filial generation [71]. This paper determined that RNA editing stress-induced modifications can be detected across several generations, adding a step of complexity on the epigenetics/epitranscriptomic factors underlying the molecular mechanisms that can lead to psychiatric diseases.

### 2.3. Antidepressant Treatment Modulate RNA Editing Activity

Due to the central role of RNA editing in the proper function of the brain and its involvement in neuropsychiatric disorders [24,52], large attention has been given to the effect of antidepressant on RNA editing modifications.

As for MDD and stress paradigms studies, the majority of the analyses correlating RNA editing and antidepressants were focused on the 5-HT2c-R (Table 2). Chronic treatment with fluoxetine, was found to increase the editing level of the A and B sites in the HI and in the striatum [72], but not in the cortex [56,72] of C57BL/6 mouse strain. Furthermore, a similar treatment performed on BALB/c mice was found to increase 5-HT2c-R editing at all sites, but not in E site, in the FC [56]. In rats, however, chronic treatment with reboxetine lead to a downregulation of 5-HT2c-R editing at E and D sites in the FC [73]; C57BL/6 mice chronically treated with the tricyclic antidepressant amitriptyline (a 5-HT2c-R antagonist) or with SB-206553 (a selective 5-HT2c-R antagonist) showed an upregulated editing level in the A and B sites in the HI and the striatum [72]. Transgenic mice expressing the 5-HT2c-R unedited INI form showed elevated sensitivity to desipramine and a significant decreased serotonin level in the nucleus accumbens (NAc), amygdala and striatum [74].

Intriguingly, in rats, maternal treatment with fluoxetine reduced 5-HT2c-R editing in the amygdala of the offspring at birth (out of 146 sites analysed), upregulated the expression of 5-HT2c-R mRNA and ADAR enzymes in PFC and rescued pre-reproductive stress modifications on 5-HT2c-R editing in the same region. Furthermore, maternal treatment with fluoxetine increased alterations of glutamate receptors editing levels between offspring of stress-exposed rats and their controls, leading to enhanced social preference in adult offspring [75].

Therefore, it is difficult to have a clear view of antidepressant modifications on 5-HT2c-R editing levels as results largely vary among antidepressant treatments, brain area and animal strains analyzed.

It has been shown that chronic treatments in rats with fluoxetine and desipramine, a tricyclic antidepressant (TCA), both normally used among the first medications for MDD, induced specific but modest effects on the expression level and RNA editing level at R/G site of GluA2 and at Q/R site for GluK2 [76]. Afterward, four weeks of treatment with antidepressants modified the R/G editing level for GluA2 with time-dependent outputs that are similar to the timing of the therapeutic effect of the antidepressants [77]. Interestingly, in a HeLa cell line modified to express half-edited Q/R GluA2 transcripts, normally fully edited, the level of editing was upregulated after treatment with seven different antidepressants [78]. Furthermore, in murine astrocytes, fluoxetine modulated GluK2 editing, glutamate-induced Ca^2+^ influx and kinase phosphorylation [79,80], indicating a cell peculiar effect for fluoxetine. This evidence indicates that RNA editing of kainate and AMPA receptors might be a potential target for antidepressant action. However, we recently reported that ketamine, a fast action antidepressant that chemically works as an N-methyl-D-aspartate (NMDA) antagonist and presents clinical antidepressant activity within hours post injection, has no effects on AMPA receptor RNA editing analyzed on CMS animal model [67] and naïve rats.

## 3. m6A RNA Modification

**Figure 3:**
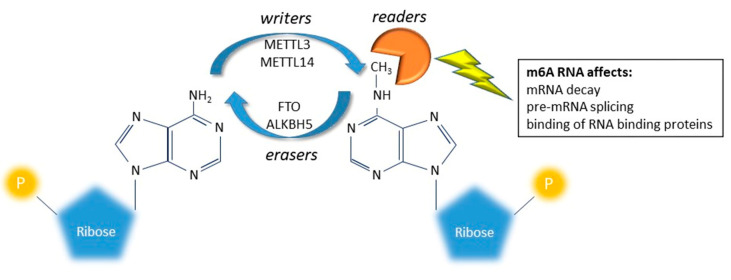
Graphical representation of m6A RNA modification. Adenosine is methylated in position N6 to form m6A by a class of enzymes globally called “writers” and demethylated by a class of enzymes called “erasers.” The two classes of enzymes work together to balance the level of m6A. The m6A message is interpreted by another class of enzymes called “readers” and plays an important role in regulating gene expression by fine modulating different aspects (processing, stability, translation etc.) of RNA molecules.

New indications suggest that several chemical modifications on mRNA, now called epitranscriptomic modifications, can affect every aspect of RNA biology, including stability, transport, splicing, and translation [7,8,9]. Among RNA changes, N6-methyladenosine (m6A) is the most prevalent in the transcriptome of eukaryotic cells and attracted high attention in recent years for its dynamic regulation [14]. Adenosine is methylated in position N6 to form m6A by a class of enzymes globally called writers (a methyltransferase complex and adapting proteins: METTL3, METTL14, WTAP, KIAA1429 and RBM15/RBM15B); m6A are then demethylated by the erasers (FTO and ALKBH5). The two classes of enzymes work together to dynamic balance the level of m6A on a specific transcript. The m6A message is interpreted by the readers (YTHDF1, F2, F3 and C1; hPUM2; hnRNPG, hnRNPC and HuR) that can affect decay, splicing and binding of RNA binding proteins (Figure 3) [7,8,9]. While recent reports started to shed light on the importance of m6A on brain development and functions [81,82,83,84,85], few reports attempted to associate this RNA modification to mood disorders. In particular, some studies tested indirectly the association between MDD and m6A RNA modification, by analyzing the association between MDD and genetic variants inside writer/eraser and reader genes. A positive association between the rs9939609 A allele, mapping in the FTO gene, and MDD was reported by Samaan et al. [86]. The same analysis, performed on MDD patients stratified by clinical subtypes, revealed that the positive association was mainly driven by the atypical MDD subtype [87]. Another study, analyzing 23 SNPs mapping inside five genes involved in the RNA modification (ALKBH5, METTL3, METTL14, WTAP and FTO), showed that in the Han population of China, the ALKBH5 gene was associated with MDD; furthermore, many SNPs inside the genes encoding the m6A system were associated with clinical symptoms of MDD such as anxiety, retardation and cognitive disturbance of MDD [88]. The reported association between m6A demethylase genes suggest that m6A RNA modification may also be involved in the development of MDD.

In addition to association studies on humans, other studies on animal models tested the effect of m6A in the brain under different conditions. Engel et al. [89] investigated the m6A and the N6, 2′-O-dimethyladenosine (m6Am) landscape (collectively called m6A/m) in the adult mouse brain after acute stress. Acute restraint stress changed the total level of m6A/m. Intriguing, variations in the m6A/m epitranscriptome decreased in PFC, but was increased in the amygdala, showing a brain region-specific pattern of modification. Moreover, similar changes in the global levels of m6A/m were reported in the same brain areas after stimulation with corticosteroid, the major stress hormone. Furthermore, using specific approaches to study single transcripts, modification of m6A amount could be observed for stress-related and synaptic related transcripts, after acute stress. The authors showed that regulation of m6A/m is impaired in MDD patients’ blood following glucocorticoid stimulation.

Finally, a very recent paper revealed that circSTAG1 is able to weaken depressive-like behaviors through the modulation of m6A/m of fatty acid amide hydrolase (FAAH). CircSTAG1 is a circular-RNA that was found downregulated in chronic unpredictable stress (CUS)-treated mice and in the blood of MDD patients. Huang et al. showed that circSTAG1 overexpression inhibited the ALKBH5 translocation into the nucleus, leading to an increased m6A methylation of FAAH mRNA and its degradation in astrocytes. This in turn determined an improvement of astrocyte functionality and a reduction of depressive-like behaviors induced by CUS [90].

This very recent data suggest that the m6A modification dynamically responds to environmental stimuli in a brain-area and gene-specific manner and that its dysregulation may contribute to stress-related disorders [91].

## 4. Conclusions and Perspective

Up to date, the vast majority of RNA editing studies in mood disorders have focused mainly on few re-coding sites in serotonin receptor 2C and glutamate receptors mRNAs. The comparison of the reported results, however, is extremely difficult due to the large heterogeneity in experimental designs (subjects analyzed, different brain areas studied, different pharmacological treatments, different technical approaches used to measure editing etc.). In addition, some studies suffer from low statistical power (due to the limited number of subjects analyzed) that impact on the reproducibility of the results.

Considering the 5-HT-2c receptor RNA editing, converging analyses reported a trend of increased editing mainly in the PFC of depressive patients that committed suicide. We might speculate that lower receptor activity, induced by higher editing in specific brain areas, could be a risk factor for suicide in MDD patients. Nevertheless, a clear and final link between 5-HT-2cR RNA editing dysregulation and MDD is still missing. It is of note that 5-HT2c-R RNA editing has been studied mainly in HI and PFC, two of the main areas involved in depressive disorders; no study at present has investigated the RNA editing level of this receptor in the nucleus accumbens, the brain area where it is expressed at the highest levels [92]. Future studies evaluating the 5-HT-2c RNA editing level in this area should be warranted in the light of the fact that the nucleus accumbens has attracted attention as a target for treatment of mood disorders [93].

As concern glutamate receptors, RNA editing has been extensively studied in many animal models of stress paradigm and after antidepressant treatments with preliminary results that, although not always concordant, seem to suggest an involvement of this mechanism in depression. Unfortunately, these data are not reflected in humans, where the absence of studies suggests a lack of positive results.

Therefore, the role of RNA editing remains extremely unexplored in the contest of MDD and a more comprehensive picture is needed to shed light on the complex pattern of modifications that underly the onset and the progression of this multifaceted disorder. Some attempts in this sense have already been reported for other neurological and psychiatric disorders with interesting results. Indeed, global analysis of RNA editing using NGS technology have shown a general dysregulation of RNA editing and ADAR activity in multifactorial diseases such as schizophrenia [31], autism, [33], Alzheimer’s [29] and amyotrophic lateral sclerosis [30]. The same approaches are needed in the contest of mood disorders; global analysis of RNA editing will broaden the number of editing sites and genes that might be dysregulated. Obtaining this data will clearly uncover the role of RNA editing in patients suffering of mood disorders. Furthermore, for human studies, genetic variations and biological and environmental variables should be carefully considered [94].

Finally, the emerging field of m6A modification open new and unanswered questions about its role in MDD. Here, we are still limited by available technologies. Future studies are needed to shed light on the epitranscriptome that dynamically regulate gene expression in our brains.

## Figures and Tables

**Table 1:** 5-HT2c RNA editing status in the brains of patients with MDD and in suicide victims.

Disease	Brain Area	Single Site Analysis	Edited Isoform	Reference
MDD	FC	not altered (sites A, C, D)	nd	[40]
Suicide victims (SCZ and MDD)	FC	↑ A, = C, ↑ D (trend)	nd	[40]
MDD	FC	= A, ↑ D (trend)	nd	[41]
Suicide victims (MDD)	FC	= A, = B,↑ E, ↓ D, ↑ C (trend)	↑ VGI, ↓ VNI	[42]
MDD	Cortex	↓ E, ↓ D, ↓ C (all trend)	not altered	[43]
Suicide victims (MDD)	PFC	nd	↑VSV	[45]
Suicide victims (MDD)	ACC	nd	↑VNI ↓INV↑ VDV	[46]
Suicide victims (MDD)	FC	nd	↑ VNI	[46]

MDD: Major Depressive Disorder; SCZ: schizophrenia; FC: frontal cortex; PFC: prefrontal cortex; ACC: anterior cingulate cortex; nd: not determined.

**Table 2:** RNA editing variations after antidepressant treatments in serotonin receptor 2C and glutamate receptors.

Animal Model	Brain Region	Treatment	Effect	Reference
Mouse C57BL/6	Striatum and HI	Chronic fluoxetine	↑A and B	[72]
Mouse C57BL/6	Cortex	Chronic fluoxetine	No Effects	[56,72]
Mouse BALB/c	FC	Chronic fluoxetine	↑A, B, C, D = E	[56]
Rat Sprague-Dawley	FC	Reboxetine	↓ D, E	[73]
Rat Sprague-Dawley	FC	Fluoxetine	Non effects	[73]
Mouse C57BL/6	Striatum and HI	Tricyclic, amitriptyline, SB-206553	↑A and B	[72]
Rat Sprague-Dawley	Amygdala of the offspring at birth	Fluoxetine	↓ A;B and C	[75]
Rat Sprague-Dawley	PFC	Fluoxetine	= GluR2 Q/R, ↑ GluR2 R/G (flip isoform only); GluR3R/G, GluR4 R/G, GluR5 Q/R, GluR6 I/V, GluR6 Y/C (no alteration) ↓GluR6 Q/R	[76]
Rat Sprague-Dawley	PFC	Desipramine	↑ GluR2 R/G (flop isoform only), ↑ GluR4 R/G GluR3R/G, GluR5 Q/R, GluR6 I/V, GluR6 Y/C, GluR6 Q/R (no alteration)	[76]
Rat Sprague-Dawley	PFC	Reboxetine	= GluR2 Q/R, ↑ GluR2 R/G (flip isoform only), ↑ GluR4 R/G GluR3R/G, GluR5 Q/R, GluR6 I/V, GluR6 Y/C (no alteration) ↓GluR6 Q/R	[76]
Rat Sprague-Dawley	HI	Fluoxetine	GluR2 Q/R, GluR2 R/G, GluR3R/G, GluR4 R/G, GluR5 Q/R, GluR6 I/V, GluR6 Y/C (no alteration) ↓GluR6 Q/R	[76]
Rat Sprague-Dawley	HI	Desipramine	↓ GluR3R/G (flop isoform only) GluR2 Q/R, GluR2 R/G, GluR3R/G, GluR4 R/G, GluR5 Q/R, GluR6 I/V, GluR6 Y/C, GluR6 Q/R (no alteration)	[76]
Rat Sprague-Dawley	HI	Reboxetine	↓ GluR3R/G (flip isoform only) GluR2 Q/R, GluR2 R/G, GluR4 R/G, GluR5 Q/R, GluR6 I/V, GluR6 Y/C (no alteration) ↓GluR6 Q/R	[76]
Rat Sprague-Dawley	P/FC	Fluoxetine	↓ GluA2 R/G (flop isoform) observed after 3 weeks of treatment and washout; ↓GluA3 R/G (flip variant) observed after 3 weeks of treatment ↑ GluA4 R/G (flip isoform) observed after 3 weeks of treatment	[77]
Rat Sprague-Dawley	P/FC	Reboxetine	↓ GluA2 R/G (flop isoform) observed after 1 and 3 weeks of treatment and washout; ↑ GluA4 R/G (flip isoform) observed at all point tested	[77]
Rat Sprague-Dawley	HI	Fluoxetine/Reboxetine	↑GluA2 R/G (flip and flop isoforms) observed after 2 and 3 weeks of treatment GluA3 and GluA4 R/G sites were unchanged	[77]
TetHeLaG2m cells		Fluvoxamine, fluoxetine, paroxetine, milnacipran, reboxetine, amitriptyline, desipramine, imipramine	↑ GluR2 Q/R after incubation with each antidepressant except reboxentine	[78]
Male adult CD-1	cultured astrocytes intact brain	fluoxetine	↑ GluK2 I/V, GluK2 Y/C, GluK2 Q/R	[79]
Male and female FVB/NTg(GFAP-GFP)14Mes/J B6.Cg-Tg(Thy1-YFPH)2Jrs/J mice	cultured astrocytes	fluoxetine	↑ GluK2 I/V, GluK2 Y/C, GluK2 Q/R	[80]
Male and female FVB/NTg(GFAP-GFP)14Mes/J B6.Cg-Tg(Thy1-YFPH)2Jrs/J	cultured neurons	fluoxetine	No alteration in any GluK2 editing sites	[80]
Rat Sprague-Dawley	HI	Ketamine	No alteration in any AMPA receptor editing sites	[67]
